# HPLC and spectrophotometry methods for measuring melamine migration from melamine dishes to food simulants

**DOI:** 10.1016/j.mex.2021.101284

**Published:** 2021-02-19

**Authors:** Ehsan Haghi, Attaollah Shakoori, Mahmood Alimohammadi, Fariba Razeghi, Parisa Sadighara

**Affiliations:** a Department of Environmental Health, Food Safety Division, School of Public Health, Tehran University of Medical Sciences, Tehran, Iran; b Food Safety Center Research, Shahid Beheshti University of Medical Sciences, Tehran, Iran

**Keywords:** Melamine, HPLC, Spectrophotometry, Migration

## Abstract

Melamine is an organic-based chemical usually found as enriched white nitrogen crystals. Since melamine is used for manufacturing food dishes and containers, there are concerns about melamine migration from the dishes into foods and subsequently the human body, particularly in children.

As there are no routine techniques to measure melamine migration in food quality control laboratories in Iran, we here aimed to validate HPLC and spectrophotometry methods to measure migration of this substance. The HPLC and spectrophotometry techniques were adopted and validated. The level of melamine migration was measured in melamine ware of five brands in Iran market. Distilled water and acetic acid 3% were used as simulants. The dishes were in contact with the simulants for 90 min at 90 °C.The accuracy and precision were obtained as 94.9 and 95.3% for HPLC and 95.3% and 96.2% for spectrophotometry, respectively. Furthermore, the limit of detection (LOD) and limit of quantification (LOQ) were obtained as 145 and 435 ng/ml for HPLC, and 200 and 605 ng/ml for spectrophotometry, respectively. Our results indicated that HPLC can be a reliable method to measure low-level melamine. The spectrophotometry could also be applied as a feasible, accurate, and cost-effective method for measuring melamine in foodstuffs.

• This research has tried to adopt a method to measure melamine migration in the regions where there are no routine techniques to measure melamine migration the same as Iranian food laboratories.
• The validation results of HPLC and spectrophotometry methods showed 94.9% accuracy and 95.3% precision and 95.3 and 96.2% for spectrophotometry respectively which were reliable.
• HPLC can be a reliable method to measure low-level melamine. The spectrophotometry could also be applied as a feasible, accurate, and cost-effective method for measuring melamine in foodstuffs.

## Graphical abstract

**Figure:**
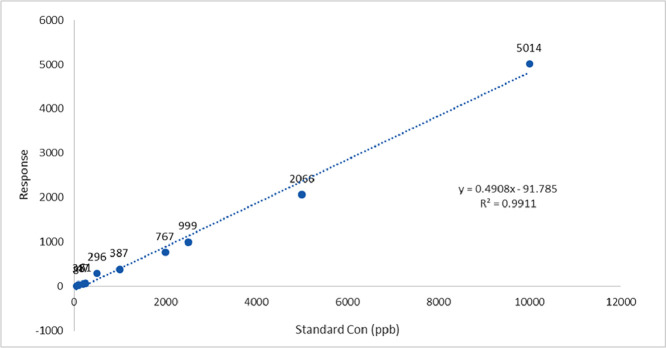

## Specifications table

**Table:** 

Subject Area:	•Engineering •Environmental Science
More specific subject area:	Food safety
Method name:	HPLC and Spectrophotometry methods
Name and reference of original method:	Rima J AK, El Omar F. A sensitive spectrofluorimetric method for the quantification of melamine residue in milk powder using the Mannich reaction in aqueous solutions. Talanta. 2013; 116:277–82. doi: 10.1016/j.talanta. 2013.05. 035. The method was based on the complexion of melamine with a mixture of formaldehyde and chemicals including a ketone group, as described by the Mannich reaction. 1,3-Diphenylpropane-1,3‑dione (DPPD) was tested as a ketone compound. We used uranin as a ketone compound. It is the difference between our method and theirs. CEN.(The European Committee for Standardization) Materials and articles in contact with foodstuffs — plastic substances subject to limitation —Part 27: Determination of 2,4,6-triamino-1,3,5-triazine in food simulants. . Brussels:CEN. 2005;CEN/TS 13,130–27 The European Committee method for articles in contact with foodstuffs — plastic substances subject was used.
Resource availability:	https://www.ncbi.nlm.nih.gov/pubmed/24,148,404

## Method details

Melamine wares were purchased from five brands of food dishes in Iran market. Distilled water and acetic acid (3%) were used as stimulants for measuring melamine migration. The dishes were in contact with the simulants for 90 min at 90 °C. The HPLC and spectrophotometry techniques were used to determine melamine migration. The study was approved by the Ethics Committee of Research of Tehran University of Medical Sciences.

### Migration measurement by HPLC

The CEN standard (The European Committee for Standardization) technique with certain modifications was used.

The modified technique represented lower costs, as well as higher accuracy and precision. These modifications included using 25% sodium phosphate buffer instead of 75% acetonitrile solution. The original mobile phase needs pH adjustments and the desorption of solution gases. This is while the modified mobile phase is a simple and cheap reagent applicable by using the distilled water: ethanol (30:70) solution. Moreover, the210nm wavelength was used instead of 220 nm to provide sharper and more transparent peaks. A cheaper XBD-C18 column was used instead of the GOA column to reduce the costs of the analyses.

The HPLC device has a diode-array detector (DAD) (infinity 1260 model) and a column of XBD-C18 (4.6 × 250 mm 5-micro). The particles size was 5 μm, the injection volume was 100 μl, the mobile phase was 30% water + 70% ethanol and the flow rate was 1 ml/min. Finally, melamine concentration was determined by reading the absorbance at 220 nm [1].

### Instruments and materials

HPLC device (Agilent Technologies, USA), ultrasonic instrument (Elma, Germany), micro-scale (µg) digital balance (Sartorius, Germany), sampler (Eppendorf, Germany), HPLC quality deionized water (multi-Q filter system), Melamine (Sigma, Germany, Lot number: 1422105v), Acetonitrile HPLC Grade (Merck, Germany), pure ethanol (Merck, Germany).

### Preparation of solutions and calibration curve

The melamine standard solution was used to prepare a serial dilution of 12 different concentrations (0. 5 to 10 μg/ ml) and draw the calibration curve. The solution of deionized distilled water and ethanol (30:70) was used as the mobile phase to be injected into the HPLC column. At first, a 30-min pilot injection was administered to determine the accurate retention times. Afterwards, 10 different melamine concentrations along with deionized distilled water were injected in triplicate to prepare the calibration curve using MS Excel 2013.

### Validation of the HPLC procedure

The offered protocol and the analytical methods including the limit of detection (LOD) and limit of quantification (LOQ), as well as the linearity, accuracy (% recovery), and precision (% RSD) of the procedure were validated according to the International Conference of Coordinated Instruction [2]. For validating linearity 6 different concentrations of melamine standard solution (0.5, 1, 2, 2.5, 5 and 10 μg/ml) and for accuracy and precision 3 concentrations (1, 2.5 and 10 μg/ml) were prepared from stock solution. The 3 replications in a day on 3 consecutive days were used to check for precision and accuracy.

Accuracy = (measured value – accepted value)/accepted value * 100

### Preparation of the samples

First, the sample melamine bowls were completely washed with distilled water and dried in the oven (30 °C). The bowls were then filled with simulants up to a distance of 1 cm from the edge. The bowls were heated to 90 °C. In order to preserve the temperature of the simulant during its contact with the dishes, the bowls were placed in the oven to reach the desired temperature. After that, the simulant was taken out, transferred into glass tubes, and kept in refrigerator until the injection time.

In the next step, 1 ml of the sample was transferred into a capped test tube. Thereafter, 9 ml acetonitrile was added to the test tube. Nitrogen was fully dried at 25 °C by gas flow. After the sample was fully dried, 1 ml of the mobile phase was added to the sample. After a full shake, the sample was transferred into a specific vial and injected into the HPLC device following the below instructions: flow rate: 1 ml/min, injection volume: 100 μl, the wavelength: 220 nm, mobile phase: water+ ethanol (30:70 ratio), and retention time: 6 minutes.

## Measurement of melamine migration by spectrophotometry

For this purpose, the UV/VIS spectrophotometer was used. The melamine migration was measured based on the formation of the melamine, formaldehyde, and a ketone group (Uranin) complex (i.e. Mannich reaction) [3].

### Instruments and materials

We used UV/VIS spectrophotometer (PerkinElmer, USA), micro gravity detection scale (Sartorius, Germany), ultrasonic (Elma, Germany), sampler (Eppendorf, Germany), Deionized distilled water (multi-Q filter system), Melamine (Sigma, Germany, Lot number: 1422105v), Uranin (C20H10Na2O5) (Sigma, Germany), and formaldehyde solution (Merck, Germany).

### Preparation of solutions and calibration curve

Initially, the stock solutions (6.3 μg/ml) for each of melamine and Uranin were prepared. Pure formaldehyde was used as another component of the mentioned complex [3].

Ten different concentrations (0.05 to 2 μg/ml) were prepared from the melamine stock solution. For developing the complex, 0.5 ml Uranin (as the ketone) and l ml formaldehyde were added. Then deionized distilled water was added to reach the 5 ml volume (Table 1). The final Uranin concentration was 0.63 μg/ml, and the final melamine concentrations varied from 0.063 to 2.52 μg/ml. After this, the calibration curve was drawn by reading the absorptions at 210 nm by spectrophotometry. The experiment was repeated three times for each concentration (The melamine–Uranin–formaldehyde complex showed the peak absorption at the wavelength of 210 nm which was applied to refine the samples and quantify melamine migration).

**Table 1:** the concentrations of the melamine–Uranin–formaldehyde complex applied to prepare the calibration curve.

Voluhme of melamine (6.3 μg/mL) (ml)	Melamine Con (ng/ml)	Volume of Uranin (6.3 μg/mL) (ml)	Volume of formaldehyde (ml)	Volume of Water (ml)
**0.05**	**63**	**0.5**	**1**	3.45
**0.2**	**252**	**0.5**	**1**	3.3
**0.25**	**315**	**0.5**	**1**	3.25
**0.5**	**630**	**0.5**	**1**	3
**0.75**	**945**	**0.5**	**1**	2.75
**1**	**1260**	**0.5**	**1**	2.5
**1.25**	**1575**	**0.5**	**1**	2.25
**1.5**	**1890**	**0.5**	**1**	2
**1.75**	**2205**	**0.5**	**1**	1.75
**2**	**2520**	**0.5**	**1**	1.5

### Validation of the spectrophotometry

This was performed based on the protocol presented by the International Conference of Coordinated Instruction. The analytical methods including the calculations of LOD and LOQ, linearity, accuracy (% recovery), and precision (% RSD) were validated [4]; Ten different concentrations (630–2520 ppb) from standard melamine were prepared. The 3 replications in a day on 3 consecutive days were used to check for precision and accuracy (Table 4,5, and 6).

All the parameters mentioned in the HPLC section were also calculated [4].

LOD = 3.3 (SD of intercept/slope)

LOQ = 10 (SD of intercept/slope)

### Preparation of the sample and reading absorption

Similar to the protocol mentioned in the HPLC section, the samples were prepared to determine melamine migration using spectrophotometry. Initially, 3.5 ml of the sample was transferred to the test tube. Then, 0.5mlUranin and 1mlformaldehyde were added to the test tube. After well mixing, the absorption of the sample was read at 210 nm using spectrophotometry.

In order to determine the sample absorption, the melamine–Uranin–formaldehyde complex (i.e. Mannich reaction) was initially scanned in the UN range (200–400 nm). Then; the wavelength in which the complex had the maximum absorption was selected. The absorption was set to zero using a blank solution (i.e. the solution with all the existing elements in the sample except for melamine). Finally, the absorption of the sample was read at the wavelength in which the complex (M–F–U) had the maximum absorption.

## Results

The results have been described in two independent sections; HPLC and spectrophotometry.***3–1- HPLC Analysis***

**Fig. 1:**
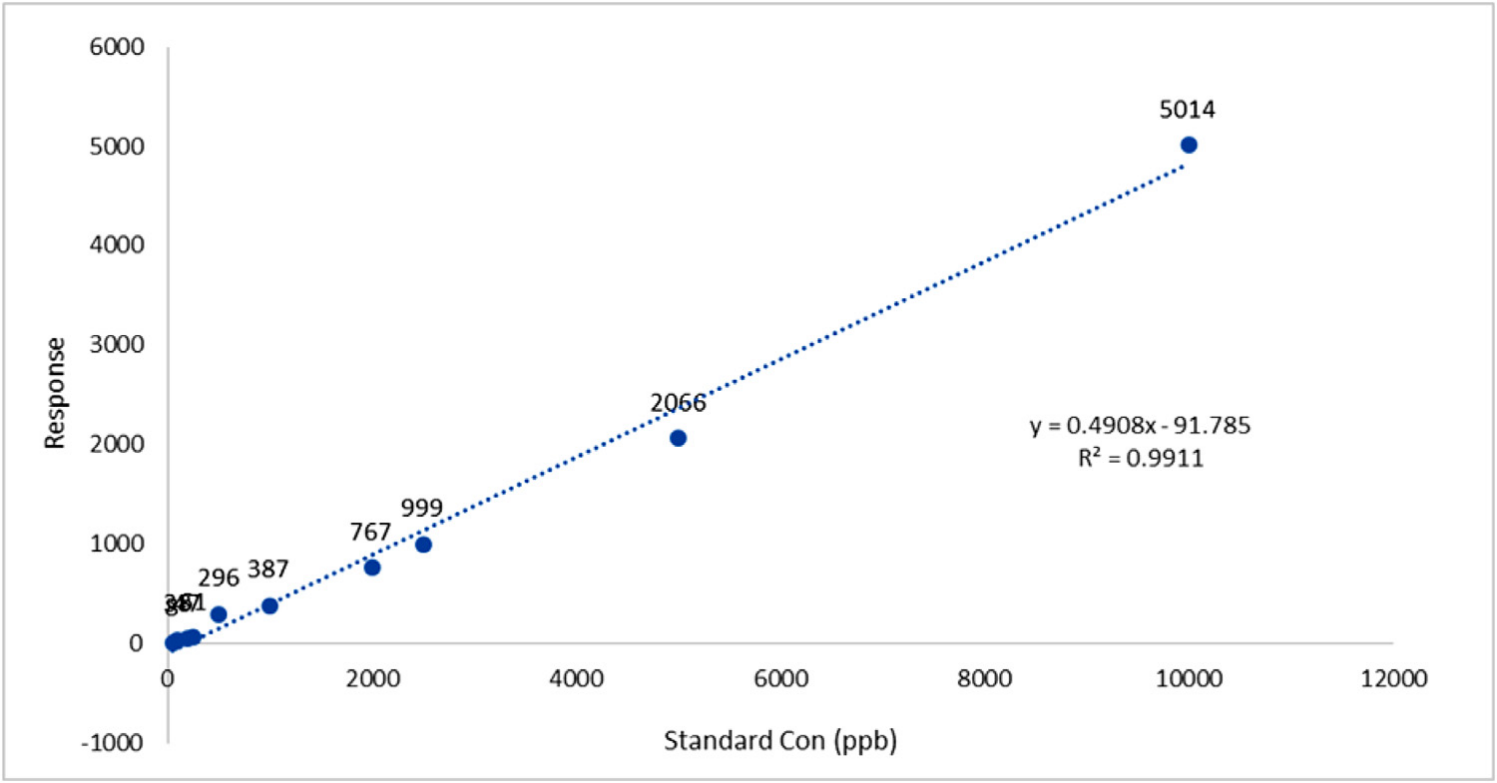
HPLC Calibration curve.

Ten different melamine concentrations (50–10,000 ng/ml) were injected into the HPLC device, and the calibration curve was drawn based on the obtained results (Fig. 1). The results of the validation phase have been shown in Table 2.

**Table 2:** the validation of the HPLC protocol presented in the current study.

Parameter	Result
Retention time	3.2 min
Accuracy (% Recovery)	94.9%
Precision	95.3%
Slope	0. 49
Intercept	−91.7
Linearity range (ng/ml)	50 - 10,000
Standard equation regression	*y* = 0.4908x - 91.785
Correlation Coefficient	R² = 0.9911
LOD (ng/ml)	145
LOQ (ng/ml)	435

### Measurement of melamine migration of samples

The melamine migration rates of five brands of melamine dishes were measured based on three repetitions, and finally the average level of the migration was reported (Table 4).

### Spectrophotometry analysis

No absorption capacity was recorded for the standard melamine solution at UV 200–400 nm. In addition, no absorption spectrums were observed for isolated formaldehyde and Uranin solutions. However; the combined solution of Uranin (0.5 ml) and formaldehyde (1 ml) revealed an absorption at 205 nm. The melamine–Uranin–formaldehyde complex showed a peak absorption at the wavelength of 210 nm which was applied to refine the samples and quantify melamine migration.

## Preparation of calibration curve and validation of the spectrophotometry technique

**Fig. 2:**
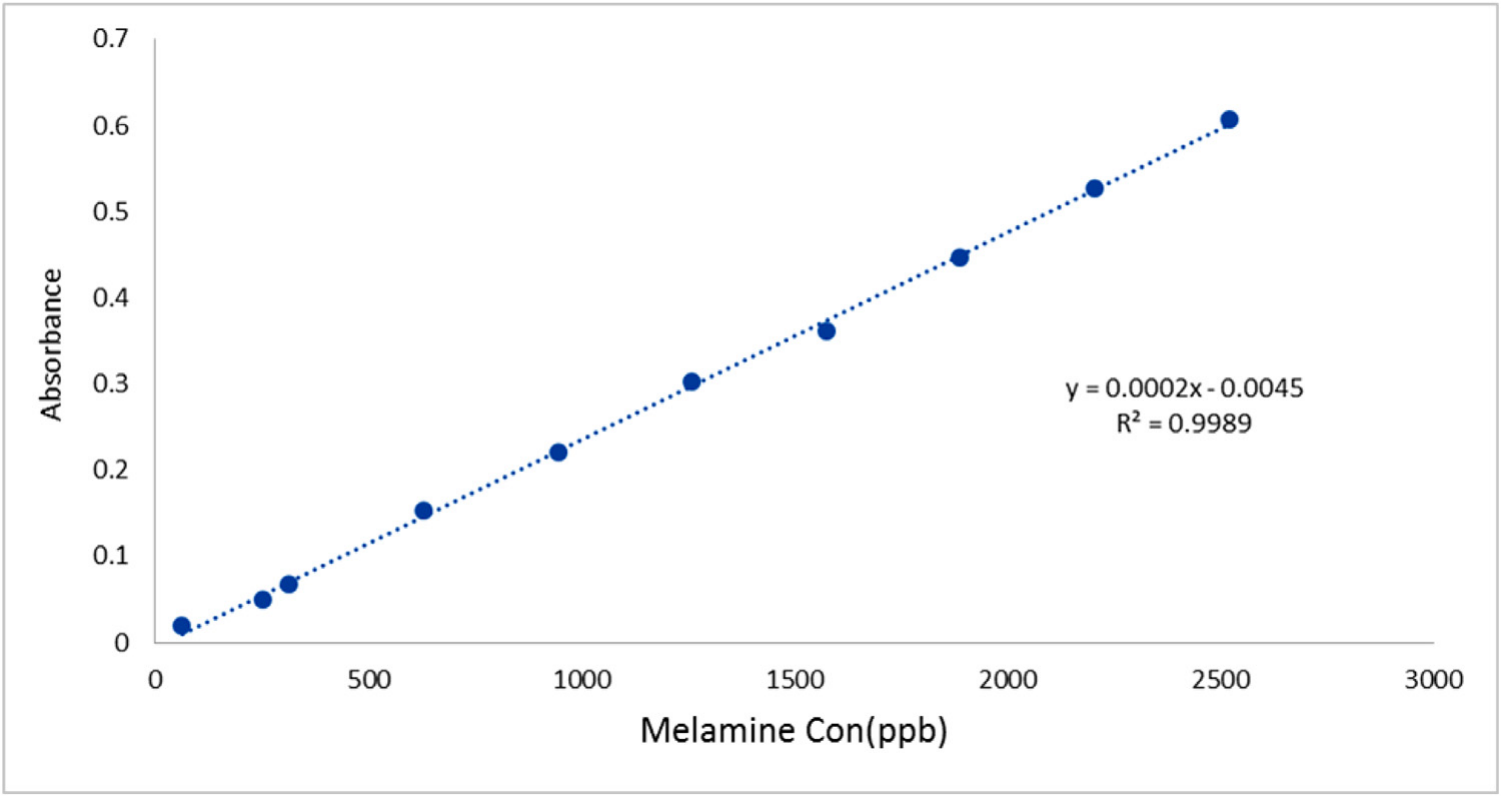
Calibration curve of the melamine–Uranin–formaldehyde complex.

The calibration curve was prepared after reading the absorptions of 10 MUF complexes solutions with different concentrations of melamine (0.063_2.52 μg/ml) at 210 nm (Fig. 2). The results of the validation of spectrophotometry technique have been presented in Table 3.

SampleTestHPLCResult(ppb)spectrophotometeryResult(ppb)

**Table 3:** the validation of the spectrophotometry protocol presented in the current study.

Parameter	Result
Maximum absorption	210 nm
Accuracy (% Recovery)	95.3%
Precision	96.2%
Slope	0.0002
Intercept	−0.0045
Linearity range (ng/ml)	63 - 2520
Standard equation regression	*y* = 0.0002x - 0.0045
Correlation Coefficient	R² = 0.9989
SE of Intercept	0.004038571
SD of Intercept	0.012115713
LOD (ng/ml)	199.9092613
LOQ (ng/ml)	605.7856403

## Measuring melamine migration of the samples

As previously stated, melamine migration was determined in melamine dishes from five different brands available in Iran market. All the experiments were conducted in triplicate. The melamine migration rates were determined using the calibration curve and a calibration line formula. The melamine migration levels have been shown in Table 4.

**Table 4:** Melamine migration measurements by the spectrophotometry and HPLC methods.

Sample	Test	HPLC Result (ppb)	spectrophotometery Result (ppb)
Sample 1	Water 90 °C - 90 min	1609	1690
	Acetic acid (3%) 90 °C - 90 min	7552	7589
Sample 2	Water 90 °C - 90 min	1362	1359
	Acetic acid (3%) 90 °C - 90 min	3224	3207
Sample 3	Water 90 °C - 90 min	2898	2843
	Acetic acid (3%) 90 °C - 90 min	5648	5616
Sample 4	Water 90 °C - 90 min	2600	2742
	Acetic acid (3%) 90 °C - 90 min	6813	6712
Sample 5	Water 90 °C - 90 min	4792	4711
	Acetic acid (3%) 90 °C - 90 min	6323	6265

## Declaration of Competing Interest

The authors declare that they have no known competing financial interests or personal relationships that could have appeared to influence the work reported in this paper.

## Appendix A Supplementary materials

Supplementary Data S1 Supplementary Raw Research Data. This is open data under the CC BY license http://creativecommons.org/licenses/by/4.0/ Supplementary Data S1
