# Supplementary material for: HPLC and spectrophotometry methods for measuring melamine migration from melamine dishes to food simulants
Source: MethodsX. 2021 Feb 19;8:101284. doi: 10.1016/j.mex.2021.101284 (PMC8374290; doi:10.1016/j.mex.2021.101284)
Supplement: Supplementary Data S1 — Supplementary Raw Research Data. This is open data under the CC BY license http://creativecommons.org/licenses/by/4.0/ [file mmc1.docx]

Table 1 Precision (% RSD) and accuracy (% recovery) of the HPLC method

| **RSD (%)** | **SD (ppb)** | **Mean Rcovery (%)** | **Rcovery (%)** | **Mean (ppb)** | **Spike (ppb)** | **Day 1** |
| --- | --- | --- | --- | --- | --- | --- |
| 18.5217 | 19.1017 | 103.1314846 | 100.9526504 | 1009.5265 | 1000 |  |
|  |  |  | 104.5180155 | 1045.1802 | 1000 |  |
|  |  |  | 103.923788 | 1039.2379 | 1000 |  |
| 13.5201 | 16.1323 | 119.3208829 | 119.6113941 | 2990.2849 | 2500 |  |
|  |  |  | 118.5813997 | 2964.535 | 2500 |  |
|  |  |  | 119.7698547 | 2994.2464 | 2500 |  |
| 10.3749 | 11.5371 | 111.2019745 | 111.0743256 | 11107.433 | 10000 |  |
|  |  |  | 111.2327863 | 1123.279 | 10000 |  |
|  |  |  | 111.2988115 | 11129.881 | 10000 |  |

Table 2 Precision, accuracy and recovery of the HPLC method (day 2)

| **RSD (%)** | **SD (ppb)** | **Mean Rcovery (%)** | **Rcovery (%)** | **Mean (ppb)** | **Spike (ppb)** | **Day 2** |
| --- | --- | --- | --- | --- | --- | --- |
| 15.873 | 16.213 | 102.141 | 100.754 | 1007.545 | 1000 |  |
|  |  |  | 101.744 | 1017.449 | 1000 |  |
|  |  |  | 103.923 | 1039.237 | 1000 |  |
| 13.489 | 14.866 | 110.209 | 110.816 | 2770.420 | 2500 |  |
|  |  |  | 109.628 | 2740.709 | 2500 |  |
|  |  |  | 110.182 | 2745.574 | 2500 |  |
| 19.823 | 19.840 | 100.087 | 100.100 | 10010.092 | 10000 |  |
|  |  |  | 100.279 | 10027.919 | 10000 |  |
|  |  |  | 99.883 | 9988.304 | 10000 |  |

Table 3 Precision, accuracy and recovery of the HPLC method

| **RSD (%)** | **SD (ppb)** | **Mean Rcovery (%)** | **Rcovery (%)** | **Mean (ppb)** | **Spike (ppb)** | **Day 3** |
| --- | --- | --- | --- | --- | --- | --- |
| 18.594 | 19.238 | 103.461 | 101.348 | 1013.488 | 1000 |  |
|  |  |  | 103.923 | 1039.237 | 1000 |  |
|  |  |  | 105.112 | 1051.122 | 1000 |  |
| 11.097 | 11.263 | 101.494 | 101.626 | 2540.652 | 2500 |  |
|  |  |  | 100.992 | 2524.806 | 2500 |  |
|  |  |  | 101.863 | 2546.595 | 2500 |  |
| 16.115 | 18.271 | 116.171 | 116.026 | 11602.622 | 10000 |  |
|  |  |  | 116.382 | 11638.276 | 10000 |  |
|  |  |  | 116.105 | 11610.545 | 10000 |  |

Table 4 Precision (% RSD) and accuracy (% recovery) of the **spectrophotometry** method

| **RSD (%)** | **SD (ppb)** | **Mean Rcovery (%)** | **Rcovery (%)** | **Mean (ppb)** | **Sample(ppb)** | **1 day** |
| --- | --- | --- | --- | --- | --- | --- |
| 7.510 | 7.637 | 101.693 | 101.428 | 639 | 630 |  |
|  |  |  | 103.015 | 649 | 630 |  |
|  |  |  | 100.634 | 634 | 630 |  |
| 6.514 | 7. 526 | 107.460 | 108.015 | 1361 | 1260 |  |
|  |  |  | 107.460 | 1354 | 1260 |  |
|  |  |  | 106.904 | 1347 | 1260 |  |
| 5.744 | 6.208 | 104.444 | 104.444 | 2632 | 2520 |  |
|  |  |  | 104.206 | 2626 | 2520 |  |
|  |  |  | 104.682 | 2638 | 2520 |  |
| 6.580 | 6.879 | 104.532 | 104.532 |  |  |  |

Table 5 Precision (% RSD) and accuracy (% recovery) of the spectrophotometry method (day 1)

| **RSD (%)** | **SD (ppb)** | **Mean Rcovery (%)** | **Rcovery (%)** | **Mean (ppb)** | **Sample(ppb)** | **2day** |
| --- | --- | --- | --- | --- | --- | --- |
| 6.338 | 6.506 | 102.645 | 102.698 | 647 | 630 |  |
|  |  |  | 101.587 | 640 | 630 |  |
|  |  |  | 103.650 | 653 | 630 |  |
| 5.929 | 6. 640 | 101.190 | 101.190 | 1275 | 1260 |  |
|  |  |  | 100.714 | 1269 | 1260 |  |
|  |  |  | 101.666 | 1281 | 1260 |  |
| 4.744 | 5.033 | 106.084 | 105.873 | 2668 | 2520 |  |
|  |  |  | 106.269 | 2678 | 2520 |  |
|  |  |  | 106.111 | 2674 | 2520 |  |
| 8.489 | 8.769 | 103.306 |  |  |  |  |

Table 6 Precision (% RSD) and accuracy (% recovery) of the **spectrophotometry** Method

| **RSD (%)** | **SD (ppb)** | **Mean Rcovery (%)** | **Rcovery (%)** | **Mean (ppb)** | **Sample(ppb)** | **day 3** |
| --- | --- | --- | --- | --- | --- | --- |
| \| 7.104 \| \| --- \| \|  \| \|  \| | 7.371 | 103.756 | 102.857 | 648 | 630 |  |
|  |  |  | 103.333 | 651 | 630 |  |
|  |  |  | 105.079 | 662 | 630 |  |
| 5.510 | 6.027 | 109.391 | 109.444 | 1379 | 1260 |  |
|  |  |  | 109.841 | 1384 | 1260 |  |
|  |  |  | 108.888 | 1372 | 1260 |  |
| 6.277 | 6.658 | 106.058 | 105.992 | 2671 | 2520 |  |
|  |  |  | 105.833 | 2667 | 2520 |  |
|  |  |  | 106.349 | 2680 | 2520 |  |
| 6.283 | 6.685 | 106.402 |  |  |  |  |
